# Impact of HIV Infection on the Clinical Presentation and Survival of Non-Hodgkin Lymphoma: A Prospective Observational Study From Botswana

**DOI:** 10.1200/JGO.17.00084

**Published:** 2018-04-02

**Authors:** Michael G. Milligan, Elizabeth Bigger, Jeremy S. Abramson, Aliyah R. Sohani, Musimar Zola, Mukendi K.A. Kayembe, Heluf Medhin, Gita Suneja, Shahin Lockman, Bruce A. Chabner, Scott L. Dryden-Peterson

**Affiliations:** **Michael G. Milligan**, **Jeremy S. Abramson**, **Aliyah R. Sohani**, **Shahin Lockman**, **Bruce A. Chabner**, and **Scott L. Dryden-Peterson**, Harvard Medical School; **Elizabeth Bigger**, **Jeremy S. Abramson**, and **Aliyah R. Sohani**, Massachusetts General Hospital; **Shahin Lockman** and **Scott L. Dryden-Peterson**, Brigham and Women’s Hospital and Harvard T.H. Chan School of Public Health, Boston, MA; **Michael G. Milligan**, **Elizabeth Bigger**, **Shahin Lockman**, **Bruce A. Chabner**, and **Scott L. Dryden-Peterson**, Botswana Harvard AIDS Institute Partnership; Musimar Zola, Princess Marina Hospital; **Mukendi K.A. Kayembe** and **Heluf Medhin**, Botswana Ministry of Health, Gaborone, Botswana; and **Gita Suneja**, Duke University, Durham, NC.

## Abstract

**Purpose:**

Botswana has a high prevalence of HIV infection. Currently, there are few data regarding the sociodemographic factors, clinical characteristics, and outcomes of non-Hodgkin lymphoma (NHL)—an AIDS-defining cancer—in the country.

**Patients and Methods:**

This study used a prospective cancer registry to identify patients with a new diagnosis of NHL reporting for specialty cancer care at three hospitals in Botswana between October 2010 and August 2016. Treatment patterns and clinical outcomes were analyzed.

**Results:**

One hundred four patients with a new diagnosis of NHL were enrolled in this study, 72% of whom had HIV infection. Compared with patients not infected with HIV, patients infected with HIV were younger (median age, 53.9 *v* 39.1 years; *P* = .001) and more likely to present with an aggressive subtype of NHL (65.5% *v* 84.0%; *P* = .008). All patients infected with HIV received combined antiretroviral therapy throughout the course of the study, and similar chemotherapeutic regimens were recommended for all patients, regardless of subtype or HIV status (six to eight cycles of cyclophosphamide, doxorubicin, vincristine, and prednisone; or cyclophosphamide, doxorubicin, vincristine, and prednisone plus rituximab). There was no difference in 1-year mortality among patients not infected with HIV and patients infected with HIV (unadjusted analysis, 52.9% *v* 37.1%; hazard ratio [HR], 0.73; *P* = .33; adjusted analysis, HR, 0.57; *P* = .14). However, when compared with a cohort of patients in the United States matched by subtype, stage, age, sex, and race, patients in Botswana fared worse (1-year mortality, 22.8% *v* 46.3%; HR, 1.89; *P* = .001).

**Conclusion:**

Among patients with NHL reporting for specialty cancer care in Botswana, there is no association between HIV status and 1-year survival.

## INTRODUCTION

A second epidemic of cancer has emerged in the setting of HIV and AIDS.^1,2^ Worldwide, patients with HIV are more likely to develop cancer than those without HIV^3^ and face higher rates of mortality.^4^ HIV infection predisposes to many cancers, including the AIDS-defining cancers: Kaposi sarcoma, cervical cancer, and non-Hodgkin lymphoma (NHL).^5^

Non-Hodgkin lymphoma, specifically, represents a major source of cancer-related mortality among patients infected with HIV.^6^ Since the introduction of combined antiretroviral therapy (ART) in the developed world, survival among patients infected with HIV has dramatically improved. However, the effect of ART on the incidence and mortality of AIDS-related NHL has been debated.^7-9^

Recently, many resource-limited nations in southern Africa have adopted nationwide ART programs. Botswana, a middle-income nation with an adult HIV prevalence of 22%,^10^ implemented a comprehensive HIV treatment program in 2003. The program has provided ART to more than 87% of individuals infected with HIV nationwide,^11^ and, in the years since its introduction, overall HIV-associated mortality has decreased. However, Botswana’s persistently high burden of HIV has been associated with an increased incidence of lymphoma,^12^ and little is currently known about the epidemiology, treatment patterns, or outcomes of NHL in the country.

There are only limited published reports regarding the incidence and outcomes of HIV-associated NHL in similar, resource-limited African settings.^13-17^ Thus, the primary objective of this study was to characterize the current burden of NHL in Botswana. We sought to determine the impact of HIV infection on patients with NHL, describe the specific subtypes of lymphoma observed, catalog clinical presentations, evaluate responses to treatment, and analyze associated outcomes. We expect that the findings from this study will aid in the development of strategies to further improve the survival among those diagnosed with NHL in Botswana and across Africa.

## PATIENTS AND METHODS

### Study Participants

**Fig 1:**
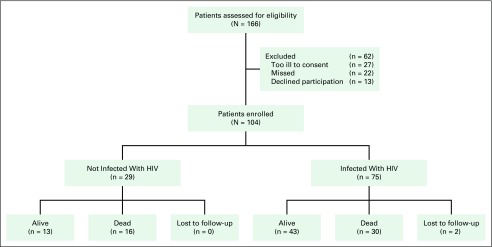
CONSORT diagram. Enrollment, retention, and vital status at the conclusion of follow-up. Enrollment registries of the Botswana Prospective Cancer Cohort include all cancers and do not differentiate by site. The number of eligible patients excluded was estimated from observed percentages of the full cohort.

Patients age 18 years or older with a new diagnosis of NHL presenting for specialized oncology care in Botswana were considered for enrollment in this study (Fig 1). All diagnoses of NHL were histologically confirmed at the Botswana National Health Laboratory before enrollment. The study was rolled out progressively, with enrollment at Princess Marina Hospital occurring from October 2010 to August 2016, Gaborone Private Hospital from November 2012 to August 2016, and Nyangabgwe Referral Hospital from January 2015 to August 2016. Patients were excluded if they were unable or refused to provide consent, if they were not citizens of Botswana, or if they had been previously treated for another cancer. On enrollment, all patients provided written documentation of their informed consent.

Patient demographics, clinical presentation, prior work-up, lymphoma subtype, comorbidities, and HIV status were abstracted from patient records and interviews. All patients not already known to be infected with HIV underwent HIV testing. For patients accessing care at public hospitals, immunohistochemistry and molecular testing were rarely available, and most cases could only be classified by histologic appearance as large-cell lymphoma, small lymphocytic lymphoma, or low-grade follicular lymphoma. A limited number of patients treated in the private sector, or by special request, had their pathology reviewed in South Africa (Lancet Laboratories, Johannesburg) with the benefit of immunohistochemistry. Cancers were staged by clinical examination, chest radiographs, and ultrasound imaging.^18^ Computed tomography, positron emission tomography, bone marrow biopsy, and CSF analysis were not routinely performed because of limited resource availability.

After enrollment, patients were contacted quarterly during clinic visits or, when unable to present to the clinic, by telephone. In the event of a patient’s death, the official cause was recorded from the death certificate, with additional context provided by family members and health care workers.

### Treatment

Standard treatment regimens were equivalent at each study site. In general, patients treated with curative intent were prescribed six to eight cycles of cyclophosphamide, doxorubicin, vincristine, and prednisone (CHOP) in 3-week intervals. Starting in 2014, treatment regimens were updated for all patients, adding rituximab to each cycle of CHOP (R-CHOP). Treatment centers in Botswana do not retain chemotherapy administration records, and thus the types of treatment, dosages, timing, and any information on toxicity or clinical response were collected from individual medical records provided by patients. A majority of the treatment records were analyzed during intensive case reviews of living patients, via patient interviews and medical record interrogation, conducted between May and August of 2014 and June and August of 2016.

HIV therapy was provided to all seropositive patients without cost by the government of Botswana. In 2012, the threshold for ART increased from 250 to 350 CD4 T cells/µL. Regardless of CD4 T-cell count, NHL is a WHO stage IV condition and represents an absolute indication for ART in Botswana.^19^ Standard first-line ART consisted of coformulated tenofovir, emtricitabine, and efavirenz.

### Data and Analysis

Lymphoma subtypes were grouped into indolent, aggressive, or unspecified categories.^20^ Survival of patients with large-cell NHL, not otherwise specified, and diffuse large B-cell lymphoma (DLBCL) were similar, and these diagnoses were grouped together as aggressive.

The primary analytic objective was to evaluate the association between HIV status and survival from the time of cancer diagnosis. Fisher’s exact test for categorical variables and *t* test for continuous variables were used to assess the significance of differences in baseline and treatment characteristics. Plots of the Kaplan-Meier estimator and log-rank tests were used to compare unadjusted survival by HIV status, and adjusted models were built using Cox proportional hazards and propensity score models, stratified by NHL behavior.^21^ Dichotomized variables known to be associated with HIV infection^22^ or survival^23^ were used to calculate the propensity score and included age, sex, performance status, cancer stage, education, and employment.

We compared survival in this cohort with patients included in the SEER Program of 20 cancer registries in the United States between 2006 and 2011. Each patient in Botswana was matched with five randomly selected patients in the SEER database on the basis of race, age, sex, NHL subtype, and stage at presentation. Analyses were performed using SAS version 9.4 (SAS; Cary, NC) and *P* values < .05 were considered significant.

## RESULTS

### Enrollment and Demographics

Between October 2010 and August 2016, 104 patients with a new diagnosis of NHL were enrolled. Seventy-five (72.2%) patients were infected with HIV. Compared with their counterparts who were not infected with HIV, patients infected with HIV were younger (median age, 53.9 *v* 39.1 years; *P* = .001) and more likely to present with an aggressive subtype of NHL (65.5% *v* 84.0%; *P* = .008). There was no relationship between HIV status and cancer stage or functional status at the time of diagnosis. Additional demographic and clinical characteristics are presented in Table 1.

**Table 1:**
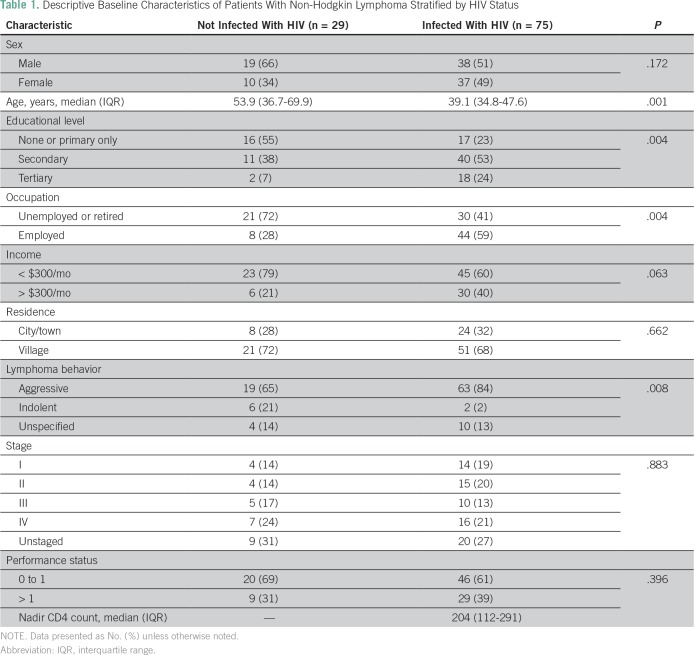
Descriptive Baseline Characteristics of Patients With Non-Hodgkin Lymphoma Stratified by HIV Status

**Fig 2:**
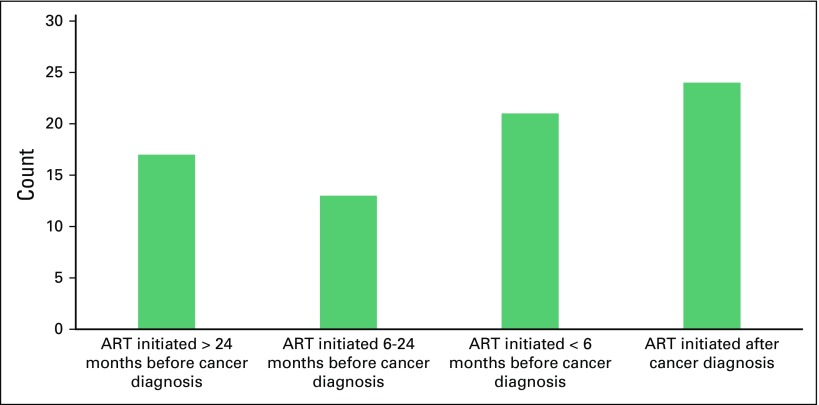
Timing of antiretroviral therapy (ART) initiation in relation to diagnosis of non-Hodgkin lymphoma (NHL) in patients infected with HIV. The largest portion (32.0%) of patients started receiving ART after the diagnosis of NHL, an absolute indication for therapy. Other indications for initiating ART are the development of other WHO stage IV diseases or CD4 counts falling below threshold. Among 75 patients infected with HIV, 28.0%, 17.3%, and 22.7% were started on ART < 6 months before their diagnosis of NHL, between 6 months and 2 years before diagnosis, or > 2 years before diagnosis, respectively.

Among patients infected with HIV, median CD4 T-cell count at the time of lymphoma diagnosis was 198 cells/µL (interquartile range [IQR], 112-291 cells/µL). The majority (64.4%) of patients who were not infected with HIV had been on ART before cancer diagnosis, with median treatment duration of 9.8 months (IQR, 1.3-28.8 months). Figure 2 presents a histogram of ART initiation relative to time of lymphoma diagnosis.

There was a long duration between the initial development of symptoms and the eventual diagnosis of NHL, amounting to 280 days on average. There was no association between the time to NHL diagnosis and HIV status (*P* = .42).

### Subtypes of Non-Hodgkin Lymphoma

Table 2 presents the subtypes of NHL most frequently diagnosed in this cohort. Among all cases, 82 (78.9%) consisted of large cells on histology. Sixty-five of these large-cell cases underwent further analysis with immunohistochemical staining, and 61 (58.7% of the entire cohort) were consistent with DLBCL. Of the other large-cell lymphomas, one was diagnosed as Burkitt’s lymphoma and three were of T-cell lineage. Indolent subtypes of NHL included small lymphocytic lymphoma (4.8%) and low-grade follicular lymphoma (2.9%) and constituted a larger proportion NHL among patients not infected with HIV as compared with patients infected with HIV (20.7% *v* 2.7%; *P* = .008).

**Table 2:**
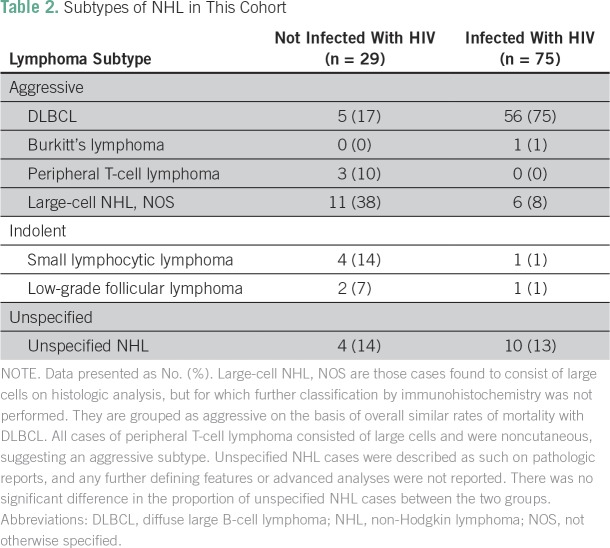
Subtypes of NHL in This Cohort

### Treatment and Toxicities

Treatment records were analyzed for 46 patients (44.2%), including 34 patients infected with HIV and 12 patients not infected with HIV. All patients were initiated on CHOP (67.4%) or R-CHOP (32.6%) and received six cycles of treatment on average. Surgical tumor excision was performed before chemotherapy in a single patient (medial maxillectomy). Seven patients with limited-stage DLBCL received radiation after completing six to eight cycles of R-CHOP.

Treatment modifications were common, with a total of 22 modifications among 20 patients. Two modifications were due to insufficient vincristine supply—vinblastine was given in its place. The remaining modifications were due to toxicities. There was one instance of treatment-related cardiac toxicity. Otherwise, 19 individual cycles of chemotherapy were delayed or cancelled because of neutropenia. There was no association between a patient’s HIV status and the likelihood of treatment toxicity (*P* = .41).

### Survival

Among the total cohort, 46 patients (44.2%) died over a median follow-up of 11.9 months (IQR, 3.9-29.6 months), including 30 patients infected with HIV (40.0% of cohort infected with HIV) and 16 patients not infected with HIV (55.2% of cohort not infected with HIV). The cause of death was reportedly due to cancer in 41 instances (89.1% of all deaths) and complications of treatment in five (10.9%). All treatment-related deaths occurred in patients with aggressive lymphomas treated solely with CHOP or R-CHOP. There was no association between the likelihood of treatment-related death and HIV status, cancer stage at diagnosis, or performance status.

**Fig 3:**
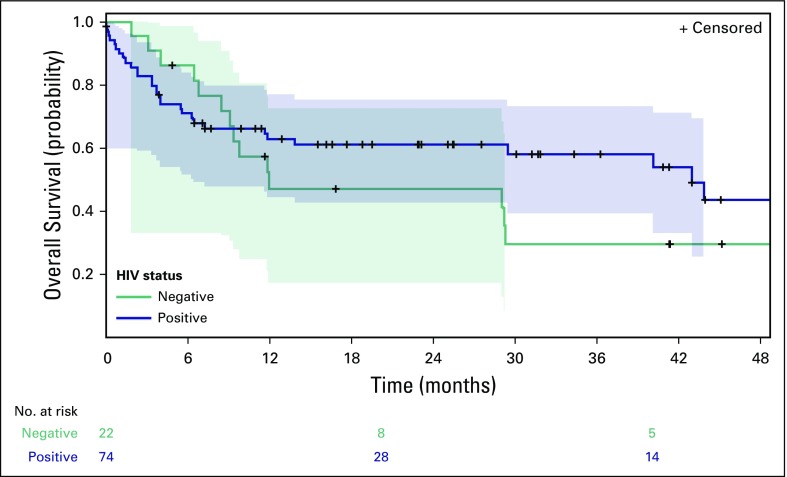
Kaplan-Meier estimated survival by HIV status. Shaded areas indicate 95% CIs. In unadjusted analysis, survival is not significantly different by HIV status (*P* = .33).

In an unadjusted model, HIV status was not associated with mortality, with patients not infected with HIV and patients infected with HIV having 1-year mortality rates of 52.9% (95% CI, 28.2% to 77.6%) and 37.1% (95% CI, 30.1% to 44.1%), respectively (HIV-infected hazard ratio [HR], 0.73; *P* = .33; Fig 3). In a model adjusted for age, sex, cancer stage, performance status, and indolent versus aggressive disease, HIV status remained unassociated with mortality (HIV-infected HR, 0.57; *P* = .14). The propensity score model also confirmed that patients infected with HIV have similar outcomes to their uninfected counterparts (HR, 0.73; *P* = .39).

**Fig 4:**
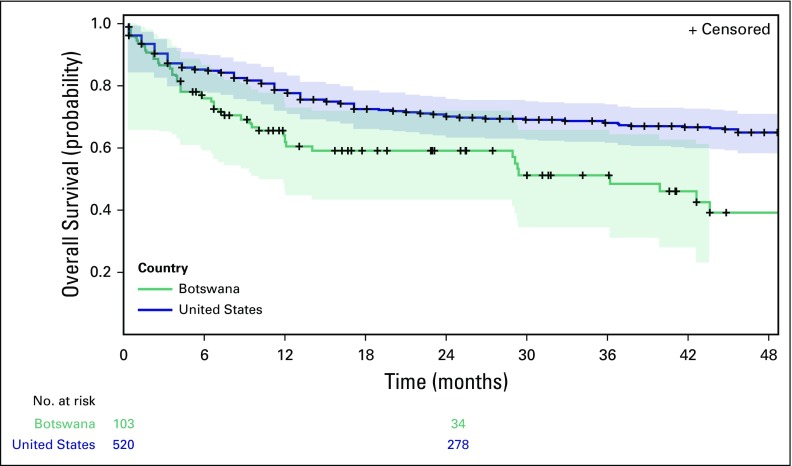
Kaplan-Meier estimated survival comparing the patients with diffuse large B-cell lymphoma in Botswana to a cohort in the United States with diffuse large B-cell lymphoma matched by race, age, sex, and stage at presentation. Patients in Botswana had a significantly lower rate of 1-year survival (*P* = .001).

Regardless of HIV status, patients in Botswana with DLBCL fared worse than the matched cohort of patients in the United States (Fig 4). Respective 1-year mortality rates were 21.8% (95% CI, 20.5% to 23.1%) in the United States and 47.2% (95% CI, 35.6% to 58.8%) in Botswana, (Botswana HR, 1.89; *P* = .001).

## DISCUSSION

Among patients with a new diagnosis of non-Hodgkin lymphoma presenting for specialized cancer care in Botswana, HIV infection is not associated with increased mortality. This prospective study is one of the first of its kind to assess the demographics and outcomes of patients with NHL, on the basis of HIV status, in a resource-limited setting in Africa. On the basis of our review of the literature, only two prior studies have explored this relationship in similar settings, in Malawi^15^ and Uganda.^24^

The prevalence of HIV infection in this cohort was markedly higher than in the general adult population of Botswana—72.1% versus 22.8%. This is consistent with the known link between HIV-associated immunosuppression and the development of NHL.^24^ Patients infected with HIV were younger and more likely to be diagnosed with an aggressive subtype of NHL, similar to data presented elsewhere.^4,25^ Many patients infected with HIV had severe levels of immunosuppression at the time of their cancer diagnosis, which should portend worse outcomes. However, along with being younger, patients infected with HIV tended to have markers of higher socioeconomic status, and younger, wealthier patients infected with HIV may have more readily accessed cancer care, better tolerated treatment, or suffered from fewer comorbidities than their counterparts who were not infected with HIV. These key differences among patients infected with HIV and patients not infected with HIV may have contributed to the similar outcomes observed in this study.

Extensive evidence has linked HIV infection to a poorer prognosis of NHL in much of the developed world.^4,24,26,27^ This relationship has been reported in other resource-limited settings as well. In Uganda, patients infected with HIV with lymphoma are twice as likely to die in the first year after NHL diagnosis as those without HIV.^28^ Patients infected with HIV receiving ART had similar outcomes to those of patients without HIV, but those not receiving ART had a nearly nine-fold increased risk of death.^29^ A prospective study evaluating the use of CHOP among patients with NHL in Malawi revealed a population with similar characteristics to the one described in this study.^15^ There was a high prevalence of HIV infection, with the majority of infected patients receiving ART. Their study found a similar 1-year mortality rate to that in Botswana (41% among those on CHOP). Although patients infected with HIV tended to experience greater treatment toxicities, they did not find a significant survival difference on the basis of HIV status.

On the basis of these reports, the comparable survival among patients infected with HIV and patients not infected with HIV in Botswana seems encouraging, if not unexpected. However, regardless of HIV status, patients in Botswana fared significantly worse than similar patients in the United States. This discrepancy likely arises from many factors. HIV infection is much more prevalent in Botswana than the United States. We did not match patients in the two countries on the basis of HIV status, and although we report no HIV-related survival difference in Botswana, patients infected with HIV with NHL in the United States have a nearly two-fold higher risk of death over a 2-year period than their counterparts who are not infected.^30^ Furthermore, the United States has a resource-intensive health care system, with rapid diagnosis and the delivery of optimal, evidence-based treatments. In Botswana, however, patients tended to present to the clinic later in the course of their disease, underwent limited diagnostic workups, and had poor access to modern treatment regimens.^22^ Thus, interventions aimed at improving the diagnosis and treatment of NHL in Botswana may lead to better outcomes in the future.

There are many challenges inherent in the diagnosis and treatment of lymphoma in Botswana. The country is sparsely populated, with the greatest prevalence of HIV—and presumably HIV-associated illnesses—concentrated in the isolated northeastern region.^20^ When presenting for care, patients with NHL typically reported vague symptoms, complicating the diagnostic work-up in an HIV-endemic region.^22^ Lymphomas may show atypical morphology and behavior in the setting of HIV,^31^ and many conditions, such as reactive lymphoid hyperplasia, HIV lymphadenopathy, and *Mycobacterium tuberculosis*^32^ infection can mimic the presentation of NHL.

Once patients were diagnosed with NHL, many of their tumors remained incompletely characterized.^18^ The cases of NHL identified in this cohort represented a large proportion of subtypes known to be associated with HIV infection, such as DLBCL.^21^ Indeed, a similar extent of subtypes has been reported in South Africa^26,33^ and among patients infected with HIV in the United States.^26^

Even after a diagnosis is made, the treatment options for patients in Botswana are limited. All patients received similar care, and it stands to reason that those with indolent subtypes may have been exposed to undue treatment toxicity, whereas those with higher-grade malignancies might have been better served by more aggressive treatments. Data have shown that treating all HIV-related cases of NHL alike leads to suboptimal outcomes.^34^ In addition, although a majority of the patients infected with HIV had been receiving ART before enrollment in this study, with 100% receiving ART after NHL diagnosis, the early initiation of ART has been shown to improve the outcomes of NHL,^10,26,33,35-37^ and the broad administration of ART to patients with HIV infection remains key for reducing the risk and mortality of NHL.

It is important to consider the results of this study in the context of its design and analysis. This study is one of the first to describe the demographics, treatments, and outcomes of patients with NHL in a resource-limited setting with a heavy burden of HIV infection. It was able to collect prospective data on a relatively large number of patients and followed them over time with minimal loss to follow-up. However, patients were enrolled in the study only after presenting for specialized cancer care. In Botswana, recent estimates have placed the incidence of NHL at roughly 5.5 cases per 100,000 person-years,^10^ substantially higher than the number of patients assessed for enrollment each year. It is likely that we were unable to characterize many patients who either never obtained a definitive diagnosis of NHL, received care in alternative settings, refused or were too ill to participate, or died before enrollment. The estimated ascertainment rate of cases was low, potentially adding bias to the study, and future studies should seek to characterize these unrepresented patients.

In addition, there were instances of missing clinical data. The ability to diagnose specific subtypes of NHL is crucial for treatment.^38^ In Botswana, limited diagnostic capacity decreases the precision of subtype classification, and advanced imaging modalities, such as computed tomography and positron emission tomography scanning, are not routinely used for staging. The prevalence of other viral infections, such as Epstein-Barr Virus and Cytomegalovirus, was not assessed, although they are highly prevalent across Africa^39^ and known to play a role in the pathogenesis of NHL.^32^ Specific treatment records were unavailable for a majority of patients, and information about treatment response or relapse was not collected. Put together, this incomplete information limited the resolution of our analysis.

Finally, the power to detect differences in survival was somewhat limited. Despite the poor survival outcomes, only 46 deaths occurred during follow-up, with 1-year mortality rates of 52.9% and 37.1% among patients not infected with HIV and patients infected with HIV, respectively. Thus, the study only had limited power to detect a difference in the mortality between patients infected with HIV and patients not infected with HIV.

In conclusion, this study enrolled patients infected with HIV and patients not infected with HIV from Botswana with a new NHL diagnosis and followed them prospectively to assess their treatment and survival over time. The prevalence of HIV infection was higher among patients with NHL than in the general population. Patients infected with HIV tended to be younger and have more aggressive subtypes of lymphoma than their counterparts who were not infected. At the time of NHL diagnosis, patients infected with HIV were likely to have severely depressed CD4 T-cell counts, independent of ART status, suggesting that HIV-associated immune dysfunction played a role in the development of NHL. Overall, 44.2% of patients died during the follow-up period, with a 1-year overall mortality rate of 41.5%. Although HIV status was not significantly associated with mortality, outcomes in Botswana lagged far behind the outcomes in more developed nations. Although many factors are likely contributing, the heavy burden of HIV, greater prevalence of aggressive subtypes, and limitations in the diagnosis and treatment of NHL likely explain much of this discrepancy. Therefore, efforts to increase the clinical awareness of lymphoma, expand the in-country diagnostic capacity, and improve the quality of treatment may lead to improvements in survival.
